# Cyclic Peptides as Novel Therapeutic Microbicides: Engineering of Human Defensin Mimetics

**DOI:** 10.3390/molecules22071217

**Published:** 2017-07-20

**Authors:** Annarita Falanga, Ersilia Nigro, Margherita Gabriella De Biasi, Aurora Daniele, Giancarlo Morelli, Stefania Galdiero, Olga Scudiero

**Affiliations:** 1 Department of Pharmacy, University of Naples Federico II, via Mezzocannone 16, 80134 Naples, Italy; annarita.falanga@unina.it (A.F.); margherita.debiasi@unina.it (M.G.D.B.); giancarlo.morelli@unina.it (G.M.); 2 Dipartimento di Scienze e Tecnologie Ambientali Biologiche Farmaceutiche, University of Campania “Luigi Vanvitelli”, Via G. Vivaldi 42, 81100 Caserta, Italy; nigro@ceinge.unina.it (E.N.); aurora.daniele@unicampania.it (A.D.); 3 CEINGE-Biotecnologie Avanzate Scarl, Via G. Salvatore 486, 80145 Napoli, Italy; 4 Dipartimento di Medicina Molecolare e Biotecnologie Mediche, Università degli Studi di Napoli “Federico II”, 80131 Napoli, Italy

**Keywords:** defensins, defensin analogs, antimicrobial drugs, cyclic peptides

## Abstract

Cyclic peptides are receiving significant attention thanks to their antimicrobial activity and high serum stability, which is useful to develop and design novel antimicrobial agents. Antimicrobial peptides appear to be key components of innate defences against bacteria, viruses, and fungi. Among the others, defensins possess a strong microbicidial activity. Defensins are cationic and amphipathic peptides with six cysteine residues connected by three disulfide bonds found in plants, insects, and mammals; they are divided in three families: α-, β-, and θ-defensins. α-Defensins are contained in the primary granules of human neutrophils; β-defensins are expressed in human epithelia; and θ-defensins are pseudo-cyclic defensins not found in humans, but in rhesus macaques. The structural diversities among the three families are reflected in a different antimicrobial action as well as in serum stability. The engineering of these peptides is an exciting opportunity to obtain more functional antimicrobial molecules highlighting their potential as therapeutic agents. The present review reports the most recent advances in the field of cyclic peptides with a specific regard to defensin analogs.

## 1. Introduction

Cyclic peptides are considered as a new force against antibiotic resistance. These molecules, due to their characteristics, are increasingly used in the design of antimicrobial molecules. Cyclic peptides are known for their remarkable stability which allows them to withstand digestion and makes them attractive for engineered tools active to combat antibiotic resistance. Cyclic peptides present several additional properties such as: the large surface area which provides a high affinity and selectivity for the targets; a limited conformational flexibility, which enhances binding properties; and low toxicity. Moreover, they have the ability to target protein-protein interactions because they are able to fill the available chemical space and may represent an alternative to small molecules. The amount of artificial cyclic bioactive peptides is constantly growing. To date, in fact, more than 40 cyclic molecules have been designed and tested; for the most part, they have been synthesized from endogenous molecules or hormones [1].

Among endogenous antimicrobial peptides are defensins. Defensins are small cysteine peptides rich in cationic residues and widely expressed from plants to vertebrate. Defensins are recognized and classified as peptides that have evolved in defense. Their activity is mainly focused on infections due to bacteria, fungi, and many enveloped and non-enveloped viruses. Defensins show common and stable characteristics that have been preserved during evolution: six (in vertebrates) to eight conserved cysteine residues and 18–45 amino acids in length.

In this review, we provide a general description of cyclic peptides and of their potential in the field of medicine with an in-depth description of engineered cyclic defensins.

## 2. Structure and Function Relationship: A Tool to Develop Antimicrobial Drugs

There are some key structural arrangements shared by most antimicrobial peptides (AMPs) [2] which allow their broad classifications into the following categories: α-helices, β-sheets, mixed structures, and non- α- or β-structures (extended). This classification may present several shortcomings, in fact, although several peptides fall within the same structural class, their modes of action may vary significantly.

Several structural parameters need to be considered in order to understand their therapeutic potential, such as major conformational rearrangements that take place on contact with membranes of microorganisms, the appropriate balance of hydrophobicity, amphipathicity, and cationicity [2,3,4,5].

The most common motif found in AMPs is the α-helix; however, many of them exist as extended or unstructured conformers and only assume well-defined α-helical and/or β-sheet-like structures upon interaction with phospholipid membranes [6,7]. Considering that the presence of the α-helix is key to promote interactions with membranes, assist membrane lysis and, thus, for the antibacterial activity, many groups have actively included in the design of potential novel antimicrobial peptides and the modification of peptide α-helical content; but this change is not always helpful because it is now well established that the antimicrobial potency is related to the inducibility of a helical conformation in a membrane mimicking environment rather than to the intrinsic helical stability. For instance, the propensities of α-helical conformation can also be proportional to the toxicity of the peptides against mammalian cells.

One common property of most AMPs is the presence of a number of positively-charged amino acids (lysine, arginine, and histidine) which determines an overall charge between +1 and +7 [8]. Positive charges are mainly involved in the interaction between the positively-charged peptides and the negatively-charged bacterial membrane surfaces through electrostatic forces. It has been demonstrated that increasing the net cationicity beyond a limit no longer results in increased antibacterial activity. In addition, also the position of the charged residues is a key factor which determines the overall antibacterial activity. Several reports describe how the presence of positive charges is key to enhance the interaction with bacterial membranes compared to zwitterionic mammalian cells, thus reducing mammalian toxicity.

As a consequence, hydrophobicity is certainly another key issue involved in the determination of the overall activity of a given AMP. It is involved in the potential interaction with bacterial membranes of different compositions and may be correlated with acquisition or loss of antibacterial specificity. The reduction in the hydrophobicity determines a reduction of mammalian cell interactions while favoring the targeting of bacterial cell membranes, as long as the peptide has sufficient positive charge.

Together with the secondary structure, charge, and hydrophobicity, amphipathicity also plays a key role; in fact it allows the peptide to insert the hydrophobic face into the membrane bilayer. Amphipathicity in AMPs usually refers to segregation into hydrophobic and hydrophilic domains and is usually a descriptor used for dissecting the role of amphipathicity in peptide antimicrobial activity; it is measured by the hydrophobic moment of peptides when already in α-helical conformation. For peptides that adopt β-sheet conformations, the amphipathicity is achieved by the organization of β-strands into separate polar and non-polar domains which are stabilized by disulfide bridges or head-to-tail cyclization which provide a high conformational rigidity in aqueous solution. In this context, also salt-bridges may play a key role as well, contributing greatly to the overall stability of the secondary structure. The polar and non-polar domains in β-strands allow antimicrobial peptides to successfully interact with target membranes and once associated with the membrane, the amphipathic nature enables membrane disruption via formation of transmembrane channels.

The length of the peptides varies greatly among AMPs. This raises the question whether or not there is an optimal chain length that is beneficial for the observed activity of AMPs. There is no definite length of a peptide sequence that is to be taken into account when one designs novel antimicrobial peptides.

A precise balance of the described properties will allow AMPs to fold into a beneficial amphipathic conformation upon interaction with cell membranes, with positive charges present on the polar face establishing electrostatic interactions with the negatively charged head groups of phospholipids. Then, the hydrophobic interactions between the nonpolar face of the peptides and the hydrophobic domain of the bilayer will allow insertion into the membrane and will determine an increased permeability and loss of barrier function of target cells. However, the therapeutic applications have been delayed by their toxicity determined by their ability to lyse also eukaryotic cells. In order to solve these problems, rational design has gained great importance and represents a major revolution in the area of development of AMPs which are more active, more specific against the pathogen of interest, less toxic at the therapeutic doses and easy to produce on an industrial scale.

Rational design is based on bio-informatic and biophysical studies which start from native AMPs as templates for the identification of physicochemical features which are key for the design of novel antibacterial agents. AMPs can be optimized by structural modifications of key regions responsible for activity and also by the obtaining of conjugates. Studies include the regular change of amino acids or other chemical modifications which allow the obtainment of peptidomimetics which structurally mimic the key binding elements of the native peptide, preserve the ability to interact with the biological target and generate the same biological activity.

Modifications of peptide structures in antimicrobial research involve backbone and/or side chain modifications such as incorporation of unnatural amino acids (d-amino acids, β-amino acids), of N-substituted glycines, chemical modification of terminal ends of peptides, shortening of the native sequence, modifications of their amphipathic character, cyclization, lipidation, etc. [9,10,11,12].

One strategy to modify and improve the antimicrobial properties is to change from l- to d-amino acids. d-Amino acids are very rare in nature and their incorporation changes the side chain and the backbone properties of the peptide. In addition, retro-inverso peptides have also been widely used, representing a reversed peptide sequences from the N- to C-terminus.

The use of β-amino acids involves a change in the backbone of the natural peptide structures without changing the side chain chemistry, i.e., the addition of one, two or three carbons along the peptide chain. N-substituted glycines determine a relocation of the side chain from the α-carbon to the nitrogen.

Modifications of the N- and C-terminus are also widely used. The amidated C-terminus of peptides affects the hydrophobic moment of synthetic peptides, enhancing interactions with the membrane and, thus, generally improving antimicrobial activity and stability. Although it is a key strategy to improve microbial death, it also improves hemolytic activity, thus requiring case-by-case studies to evaluate the pros and cons of such modifications.

We recently described design of human β-defensin analogs which are able to address pathogens under a wide range of normal and abnormal salt concentrations; in particular, a series of chimeric peptides were obtained from human β-defensin 1 and 3 (HBD1 and HBD3) which allowed also to dissect the role played by each domain in antimicrobial activity [13,14,15].

In this scenario, the assortment of post-translational modifications of marine AMPs, which can sustain physiological salt concentration and protease activity, may help in the design of AMPs with enhanced stability and efficacy for human therapeutic applications. For instance, because of the high salinity (up to 600 mM) of the marine environment, they naturally hold a greater salt resistance than those derived from other sources and their biological activities is preserved in relatively high-salt environments, such as in saliva, gastrointestinal fluid, serum, or other body fluids [16]. Post-translational modifications include: disulfide bonds, bromination, chlorination, C-terminal amidation, the presence of high contents of specific amino acids (such as phenylalanine and arginines), the modification of single amino acids (such as 3-methylisoleucine), the presence of fatty acid linked to the peptide sequence, and the presence of d-amino acids. Some are specific only to marine AMPs and other modifications are shared also with terrestrial AMPs. Bromination is observed in cathelicidins and protects the peptide from proteases in marine environment, as also evidenced by the absence of bromination of cathelicidins derived from terrestrial mammals [17]. The peptide mixinidin is the shortest marine- derived AMP and rational modification of its amino acid sequence allowed the enhancement of its therapeutic potential [18,19,20].

The obtainment of peptide conjugates, in which various substances are attached by different coupling strategies, also represents a widely used strategy to enhance antibacterial activity.

One of these strategies involves the addition of lipid moieties. In fact, natural lipopeptides are well characterized in the literature as promising antimicrobial compounds. In particular, the addition of an aliphatic chain to the N-terminus of AMPs may improve their interaction with the bacterial surface/membrane and the overall antimicrobial properties. The length of the hydrocarbon chain is key for the activities; fatty acids of different lengths (C_12_–C_20_) were introduced at the N-terminal end of AMPs and the results showed that too long acyl chains may increase aggregation and self-assembly of the conjugates. On the contrary, C_14_–C_18_ chains were able to significantly improve minimum inhibitory concentration (MIC) values. It was proposed that linkage to fatty acids can enhance the ability to adopt secondary structures when in contact with bacterial membranes.

Recently, nanomedicine has found applications also in the design of AMPs with improved activity. Surface immobilization represents an attractive contact-killing technique to further enhance their stability, range of action, and to reduce the development of resistance, proteolysis, and cytotoxicity [21]. Several kinds of nanoparticles produced from a large variety of materials and resulting in different shapes, sizes, and surfaces have been decorated with AMPs. The activity of the peptide generally seems to be enhanced when combined with nanoparticles [22,23,24,25,26,27,28,29]

Furthermore, attachment to particles can be used to avoid any dissemination of AMPs to the environment and reduce ecotoxicity [23,30].

Paramagnetic silica microparticles were surface-modified by magainin [22]. Multifunctional particles exhibiting antibacterial and magnetic properties were obtained. Such conjugates may be used to efficiently disinfect aqueous solutions, avoiding any dissemination of bactericidal substances in the environment, but also for in vivo applications in medical, cosmetic, or biomedical fields due to the possibility of directing them by magnetic fields to a localized antibacterial action [22].

Recently, cecropin-melittin AMP-decorated gold (Au) nanoparticles were coated on glass slides [24] and antibacterial activity against *Escherichia coli* and *Staphylococcus aureus* was tested. The authors report that the antibacterial activity of this conjugate was more effective in long-term exposure compared to the free AMP with no toxicity against human cells.

Star-shaped peptide polymer nanoparticles have also been recently proved to be a novel class of antimicrobial agents with superior in vitro and in vivo efficacy against Gram-negative pathogens, including multidrug-resistant species [25].

The conjugation of silver nanoparticles with AMPs is also an emerging strategy to achieve superior antimicrobial activity which exploits the antibacterial activity of both the silver nanoparticle and the peptide [31].

Interestingly, the application of AMPs for the treatment of intracellular pathogenic bacteria is limited by their in vivo instability and low penetrating ability into mammalian cells. Recently, it was reported that gold nanoparticles conjugated with DNA aptamer is able to efficiently deliver AMPs into mammalian cells allowing enhanced stability of the peptide [27].

Another key issue is represented by the prevention of the formation of biofilm infections. Coating of surfaces with AMPs may help to reduce or prevent their formation. These approaches include the use of AMPs to develop anti-adhesive surface coatings and to kill biofilm-forming cells either on contact or via controlled release (leaching surfaces). In vitro results for all these applications are very encouraging, but still further research is needed for in vivo applications [32]. Engineering biomaterial surfaces that exploit AMPs properties, offer a promising approach to prevent implant infections [33]. Yazici et al., reported of a chimeric peptide which, while presenting its antimicrobial properties, forms a robust solid-surface coating and represents a potential solution for developing infection-free surfaces by engineering implant interfaces with highly-reduced bacterial colonization property [33].

## 3. Design and Structure of Cyclic Analogs

Among innovative approaches applied in recent years to develop AMPs with enhanced activity is the rational design of cyclic peptides.

AMPs can be naturally found in cyclic conformations; they are constrained in this conformation either by disulfide cross-linkages or backbone cyclization. Cyclic AMPs have demonstrated significant antimicrobial activities against different pathogenic bacteria, but often they present poor selectivity; thus, structure-activity relationship studies are key for improving their therapeutic profiles.

Cyclic lipopeptides, such as polymyxins, daptomycin, surfactin, iturin, fengycin, paenibacterin, and pseudofactin, are also attracting significant attention [34]. Among them, daptomycin is the most prominent [35].

Tryptophan and arginine-rich cyclic hexapeptides of the type cyclo-RRRWFW present antimicrobial activity against Gram-negative and Gram-positive bacteria and low hemolytic activity [36,37]. Minimal changes in both the cationic and hydrophobic domains of the peptides produced significant reductions of antimicrobial activity and/or modifications in the mode of action; moreover, cyclic molecules demonstrated improved activity, when compared to that of the linear analog sequences.

Other studies have exploited the native structure of the gramicidin S, as a model template for synthesis of novel cyclic antimicrobial peptides [38]. The substitution of the two positively-charged lysine residues with two ornithine residues, and of the two more polar tyrosines with the two less polar aromatic phenylalanine residues, provides a cyclic peptide with enhanced activity. Other studies have dissected the effect of ring size (4–14 residues) on the antimicrobial and hemolytic activity [39]. Membrane disruption was shown only for analogs with 10 or more residues in the ring structure, whereas only one cyclic analog showed improved antibacterial specificity.

Several cyclic peptides have been approved by the Food and Drug Administration (FDA) and have entered into the market recently as antibacterial compounds (Table 1). Telavancin, dalbavancin, and oritavancin are cyclic lipoglycopeptides belonging to the same drug class as vancomycin and teicoplanin, which contain a common heptapeptidic core with five residues that constitute the main binding site for the d-Ala-d-Ala target [40]. Binding of these drugs to their target blocks the transpeptidation of peptidoglycan precursors in the bacterial cell wall [41]. The lipophilic side chain probably anchors them to the cell membrane, and/or destabilizes the bacterial membrane; moreover, its interactions with cell membranes and plasma proteins also helps in prolonging the plasma half-life. These three drugs are used for the treatment of complicated skin and skin structure infections and nosocomial pneumonia. The small variations in their structures cause subtle differences in their pharmacologic effects by fine-tuning their activities towards different bacterial strains or differing pharmacokinetic properties.

POL7080 is an antibacterial cyclic peptide specific for *Pseudomonas aeruginosa*, which was developed through multiple iterative rounds of peptide library synthesis and screening for enhanced antibacterial activity [42]. It was designed starting from protegrin I, a β-hairpin peptide with broad antibacterial activity which was attributed to its membrane lytic properties. The secondary structure was stabilized through the use of a d-Pro-l-Pro β-turn motif [43] and several rounds of amino acid substitution, yielded the 14-amino acid peptide characterized by reduced cell lysis, but with high activity and selectivity towards *P. aeruginosa*. The peptide POL7080 showed a new mode of action compared to its precursor, involving the binding to the membrane protein LptD, involved in membrane biogenesis. POL7080 is actually in Phase 2 clinical trials for the treatment of *P. aeruginosa* infections and was demonstrated to be clinically safe and tolerable.

## 4. Defensins

**Figure 1:**
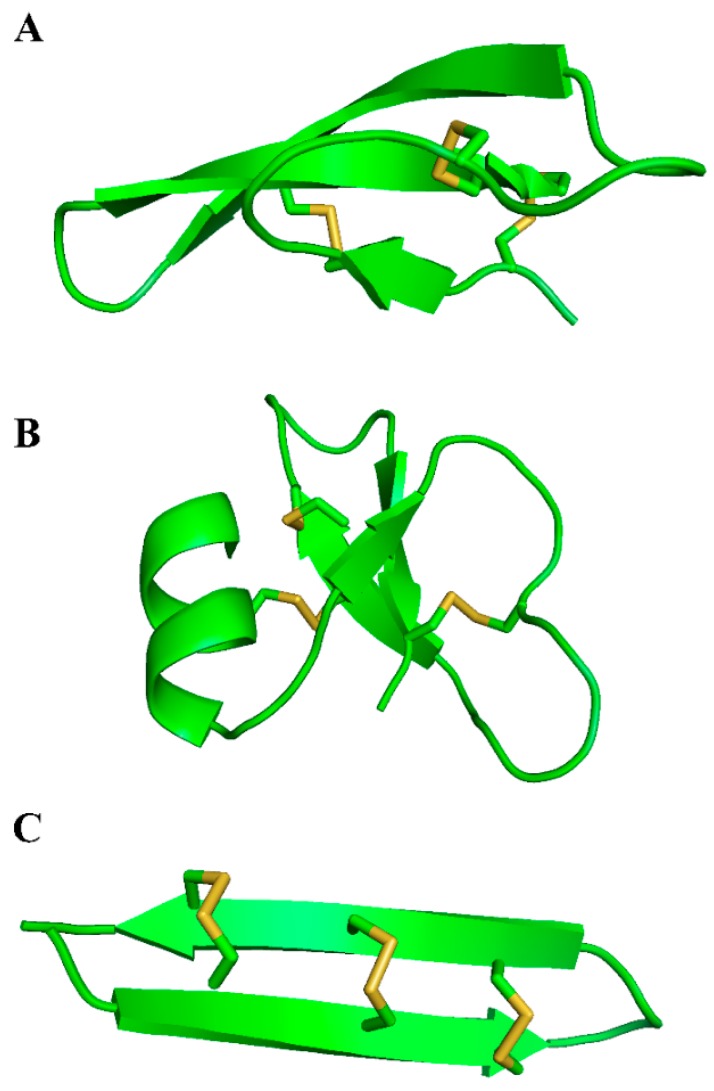
Structure of human defensins. Crystallographic structure of human α-defensin 1 (**A**); crystallographic structure of human β-defensin 1 (**B**); and the NMR-structure of θ-defensin 2 (**C**). Yellow shows the disulfide bridges.

Defensins belong to the β-sheet class of antimicrobial peptides in vertebrates [44]. They are cysteine-rich peptides, with three disulfide bridges composed of six cysteine molecules. They are divided in three subfamilies: α-, β-, and θ-defensins, according to the position of disulfide bridges (Figure 1).

### 4.1. Human α-Defensins

α-Defensins (29–35 aminoacids) are produced by promyelocytes, the neutrophil precursor cells, and in the intestinal Paneth’s cells after bacterial infection. Humans express six α-defensins, subdivided into myeloid (HNP1-4) and enteric (HD5 and HD6) peptides on the basis of both expression patterns [45]. The expression of HNP1-4 is mostly detectable in neutrophils, even though it is also possible to detect their expression in monocyte/macrophages, natural killer cells, some T cells, B cells, and immature dendritic cells (DCs). HD5 and HD6 are expressed by the intestinal Paneth cells; HD5 is present in the male and female genitourinary tracts expressed by epithelial cells.

They are produced as pre-pro-peptides of about 110 amino acid residues comprising an N-terminal domain and a pro-peptide [46]. In neutrophils, α-defensin precursors are excised by neutrophil elastase and proteinase 3. α-Defensins in the mature form are preserved in azurophilic granules. Once infection occurs pathogens are ingested into phagocytic vacuoles containing defensins. It has to be noted that α-defensins constitute more than 7% of the total protein content in neutrophils within the azurophilic granules. In the Paneth cells of the intestinal crypt, α-defensin precursors are processed extracellularly and the process is mediated by a trypsin isoform.

In both neutrophils and intestinal crypt, at basal levels, α-defensins have a very weak expression but, in response to an infection, α-defensin concentrations in blood and/or the intestine may increase by 10-fold. Paneth cell α-defensins are secreted extracellularly with a local concentration between 25 and 100 mg/mL when released [47]. It has been speculated that the level of these compounds can influence and modify the composition of the enteric microbiome. In this view, α-defensins are responsible not only for the removal of pathogens but also for the creation of a symbiosis with the normal intestinal microbiota to maintain the correct the intestinal environment.

In their secondary structure, α-defensins are linked by cysteines 1–6, 2–4, and 3–5. It is still debated whether disulfide bridges are indispensable for α-defensins activities. All of the α-defensins were shown to multimerize at least into dimers [48]. α-Defensins play a role in a very large number of cellular processes [44,49], including wound healing, angiogenesis, fertility, cancer progression, etc. Here, we will analyze only the two main functions of α-defensins that represent the starting point for the design and synthesis of engineering molecules: (1) antimicrobial action [50], and (2) chemotactic activity [51].

1. The antimicrobial mechanism of action is almost common between α- and β-defensins; several modes of action have been speculated, but it seems ascertained that the initial phase consists of the interactions between the positive charges of peptides and negative membranes of bacteria and viruses. The feature of human cell membranes being neutral guarantees the selective contact of defensins with host pathogens. Once interacted with membranes, defensins amass into the membrane of microbes and cause depolarization, which would finally induce death. The capacity to assemble in dimers, and maybe in oligomers, assists this process in determining the pores in the membranes. In addition to this well-described mechanism, both α- and β-defensins can also avoid pathogen cellular internalization by interacting with membrane/envelope glycoproteins. Specifically for α-defensins, it has been anticipated that a further antiviral mechanism exists; accordingly, α-defensins, by interacting with human cells, can shrink virus replication and transcription.
2. Thanks to their chemotactic activity, α-defensins are also considered molecules able to activate the immune system. Indeed, in vitro studies demonstrated that HNP1, HNP3, and HD-5 increase the migration of macrophages, T cells, and immature mast cells.

Beyond the attractive capacity of immune cells, α-defensins also possess a regulation function toward the production of pro-inflammatory cytokines. In fact, in response to HD-5, intestinal epithelial cells secrete interleukin-2, -8 (IL-2, IL-8), Chemokine (C–C motif) ligand 20 (CCL20), Tumor Necrosis Factor-α (TNF-α), and Interferon-γ (IFN-γ) [52].

### 4.2. Human β-Defensins

Human β-defensins (HBDs) are a family of small, highly-cationic, cysteine-rich peptides [53]. Four HBDs are predominantly expressed in epithelial cells (HBD1, HBD2, HBD3 and HBD4), although another recently-discovered HBD, HBD-9, has been seen in some districts [54]. The disulfide connectivity in HBDs are Cys1–Cys5, Cys2–Cys4, and Cys3–Cys6 (Cys, cysteine). Recently, we speculated and demonstrated an evolutionary mechanism for β-defensin development starting from a central nucleus [55,56]. Specifically, we explored the γ­core of human HBD3 demonstrating that it: (a) is the folding nucleus of HBD3; (b) folds rapidly and is stable in human serum; (c) shows antibacterial activity; (d) is responsible for HBD3 internalization in eukaryotic cells binding to CD98; (e) carries out antiviral activity against human immunodeficiency virus and herpes simplex virus; and (f) does not show toxicity to human cells [4]. These data reveal that the γ-core within HBD3 is the ancestral core of the full-length molecule and is a valid host defense peptides (HDP), per se, since it contains the most important biological features of the full-length HBD3. Notably, the small, stable scaffold of the HBD3 γ-core can be exploited to design disease-specific antimicrobial agents. Structurally, all HBDs show a high degree of similarity in their tertiary structures, despite the divergent amino acid sequences [57]. The main element of the mature peptides secondary structure is represented by three β-strands arranged in an antiparallel sheet, held together by three intramolecular disulfide bonds, formed between the six cysteines. The amino-terminal region contains a short α-helical loop (which is absent in α-defensins) which probably represents the protein region that is incorporated into cell membranes to start pathogen killing. However, it is noticed that the lack of the cysteine residues and, therefore, of the disulphide bridges, do not eliminate the antimicrobial activity of HBDs, suggesting that they can establish their structure straight in contact with pathogens.

HBDs are all expressed in epithelia, where they represent a first line of defense against microorganisms, with a preferable epithelium expression; HBD2 is robustly expressed in lung; HBD4 is greatly expressed in the stomach and testis; and HBD3 in the skin and tonsillar tissue [58]. The expression of HBD1–HBD4 is mostly present in the respiratory tract, where HBD1 shows constitutive expression and is detectable by an inducible expression of HBD2–HBD4 in response to inflammation or infection. Among the others, only in the case of HBD1, a ubiquitous expression was observed in all epithelia (but can also be upregulated); on the contrary, the other HBDs are inducible peptides, expressed at non-detectable levels in normal conditions, but subjected to a high increase during infective states. Functionally, HBDs are antimicrobial peptides active against a broad spectrum of microorganisms, such as bacteria, viruses and fungi, as well as chemotactic molecules.

In vitro assays demonstrate broad-spectrum activity of all HBDs against Gram-positive and Gram-negative bacteria, viruses (predominantly enveloped), and fungi, with minimal inhibitory concentrations in the μg mL^−1^ range [59,60].

Antimicrobial activities of most HBDs are impaired by physiological salts and divalent cations; the magnitude of inhibition depends on the defensin and its target bacteria. Importantly, HBD3 is the only peptide almost insensitive to high salt concentrations. It has been revealed that HBD2 is highly effective in killing Gram-negative enteric *E. coli* and *Pseudomonas aeruginosa* and yeast *Candida albicans* [53]. Similarly, HBD3 possesses antimicrobial activity against some pathogenic Gram-positive bacteria, such as *S. aureus* and *S. pyogenes*, as well as Gram-negative bacteria, such as *P. aeruginosa* and *E. coli* and the yeast *C. albicans* [61]. Recently, it has been reported that HBD-1, in its oxidative form, has poor in vitro antimicrobial effect [62,63]. However, reduction of disulfide-bridges makes HBD-1 a potent antimicrobial peptide against *C. albicans* and anaerobic Gram-positive commensals, such as *Bifidobacterium* and *Lactobacillus* species [62,63]. It is necessary to consider that the microbicidal capacity of HBDs is dependent on the ionic concentration of the environment in which pathogens and antimicrobial peptides interact.

In addition to the killing of microorganisms, HBDs induce the chemotaxis of dendritic cells mainly through the binding of the CCR6 receptor [64]. HBDs also promote monocyte/macrophage migration; many evidences indicate that HBD2- and HBD3-dependent chemotactic migration of human monocytes could be mediated by the C–C chemokine receptor type 2 (CCR2) [65]. HBD3 can also activate both dendritic cells and monocytes independently of CCR6 or CCR2, but via the Toll like receptor 1/2 (TLR1/2)-myeloid differentiation primary response 88 (MyD88) signaling pathway. Recently, we recognized a novel receptor on epithelial cells, CD98. We explored the intracellular fate of the native HBD3 in human epithelial cells (A549) by investigating the possible machinery responsible for the interaction of the wild-type peptide with human epithelial cell lines through confocal microscopy experiments [66]. We identified the transmembrane protein CD98 as a cell surface receptor implicated in the internalization of HBD3 in human epithelial A549 cells. CD98 is a multifunctional transmembrane protein known for its role in the innate immune response to intestinal infections determined by enteric bacterial pathogens. CD98 and HBD3 extensively co-localized on the basolateral domain of A549 cells [66]. We not only verified the direct binding of hBD3 with CD98 by fluorescence resonance energy transfer and surface plasmon resonance, but also mapped the region of interaction between the two proteins; the residues 304–414 of the CD98 protein are responsible for the binding between HBD3 and CD98, the same region known to interact with the proteins of intestinal bacteria during colonic invasion. Treatment of A549 cells with HBD3 considerably reduced CD98 expression and, on the other hand, knockdown of CD98 expression weakened HBD3 cell surface binding and internalization. Competition for bacterial binding to CD98 and downregulation of CD98 expression may represent a novel mechanism though which HBD3 exerts its antibacterial activity, i.e., competitive binding for a bacterial invasion receptor driven by electrostatic complementarily [66].

### 4.3. θ-Defensin

The θ-defensins are octadecapeptides expressed in the bone marrow and leukocytes of rhesus macaques [67]. A premature stop codon in the human gene and mRNA is responsible for the silencing of their expression in human leukocytes [67]. The structure of θ-defensins is very different from that of α-defensins and β-defensins. In fact, the mature θ-defensin peptide is a two-stranded β-sheet peptide stabilized by three disulfides, like α- and β-defensins, but the N-terminal of θ-defensins is covalently connected to their C-terminal of the molecule through peptide atoms and, therefore, they are the merely cyclized defensins [68]. They are 18 residues with three disulfide bonds in a 1–6, 2–5 and 3–4 arrangement. Six θ-defensins have been identified: retrocyclins 1, 2, and 3 and rhesus θ-defensin 1 (RTD1), RTD2, and RTD3 [69]. The genes comprise three exons and two introns. The exons encode a 76-residue pro-peptide comprising a 20-residue signal peptide, 44-residue pro-segment, two nine-residue defensin, and a three-residue tail. Although the structures and disulfide connectivities of α- and θ-defensins differ, common features in their precursor peptides suggest that similar processing mechanisms might be used. The cyclization process, through the formation of three disulfide bonds, links two nine-peptide to build a 19-defensin.

Like α- and β-defensins, θ-defensins also possess broad-spectrum microbicidal activities against bacteria, fungi, and viruses [67]. All α-, β-, and θ-defensins are antimicrobial at 0.5–5 mM; moreover, θ-defensins have the advantage of a lower sensitivity to physiological salt concentrations. The salt resistance could be due to the pseudo-cyclic structure; in fact, unstructured analogs maintain their antimicrobial activity, but at lower salt concentrations. It is notable that, apart from activity against Gram-positive and -negative bacteria, θ-defensins show a potent anti-viral activity, especially against HIV [70]. However, θ-defensins also have anti-influenza and anti-herpes activities [71,72]. The typical pseudo-cyclic structure of these peptides seems not to be critical and essential for their activity; in fact, the microbicidal activities are only slightly reduced when the peptides are de-cyclized. On the contrary, unstructured peptides lacking disulfide bonds lose their stability and are rapidly degrade in serum. In addition, the pseudo-cyclic structure guarantees θ-defensins a very high stability to several enzymes in biological fluids, a characteristic that is very important in the case of high concentrations of proteases in tissues characterized by inflammation. Starting from this observation, a number of cyclic defensin analogs have been designed and developed with the aim of finding microbicidal drugs with a great pharmacological potency and a large stability/resistance to degradation. In particular, among the others, two functional features of θ-defensins have garnered the most interest for the design and improvement of drugs: the potent anti-HIV action and the stability.

We will analyze the efforts made and the state of the art about artificially-cyclized peptides designed and synthetized to take advantages from the structural characteristics of natural defensins.

### 4.4. De Novo Design of Cyclic Peptides Starting from Defensins

**Figure 2:**
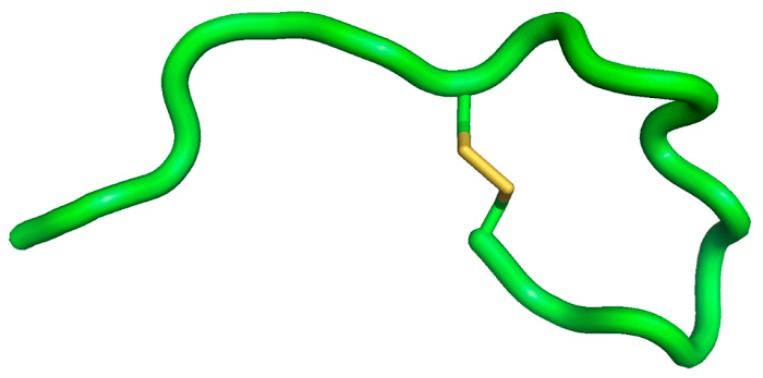
NMR-structure of AMC. Yellow shows the disulfide bridge.

Our group has recently designed a pseudo-cyclic 17-amino acid β-defensin analog with only one disulfide bond which was inspired by cyclic θ-defensins [15]. This molecule (AMC) combines the internal hydrophobic domain of HBD1 and the C-terminal charged region of HBD3. (Figure 2) The novel peptide was shown to retain the antimicrobial activities against Gram-positive and Gram-negative bacteria of the parent HBD1 and HBD3, to be considerably more stable in human serum. Taken together, these data suggest that complicated and difficult to obtain AMPs can be used as templates to design smaller antibacterial molecules.

In line with those studies are the subsequent results on peptide analogs to AMC which showed conserved antibacterial activity [55]. In fact, we further analyzed the β-defensin structure, keeping in mind the observation by Yount and Yeaman that the ‘γ-core’ is a common structural signature, which exists in virtually all host defense peptides stabilized by multiple cystine bridges [73]. The γ-core motif of HBD3 is part of a β-hairpin enclosed by the native disulfide Cys and corresponded to one of the domains identified previously by our group for its activity [13,14,15]. Consistent with the hypothesis, the obtained cyclic peptides displayed potent antibacterial activity against Gram-negative and Gram-positive bacteria, as well as antiviral activity against HIV and Herpes Symplex Virus (HSV) [55].

## 5. Conclusions

**Figure 3:**
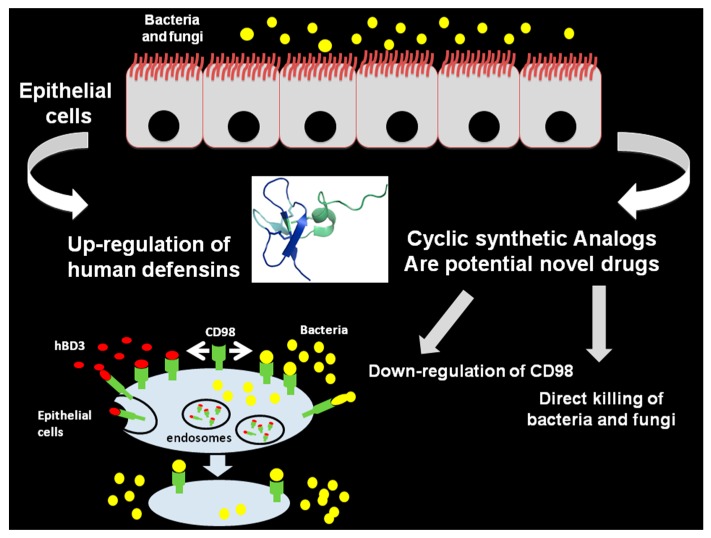
Inhibition of bacterial infections on epithelial cells by human β-defensins and potential cyclic analogs. The schematic representation shows the crosstalk between bacteria and peptides. Antimicrobial activities of peptides include the direct killing and down-regulation of CD98.

The high stability, the reduced cost of synthesis, the low toxicity and, more often, the increased activity make cyclic peptides attractive molecules to design and develop novel drugs. In addition, the large surface area and the reduced conformational flexibility generally guarantee high affinity and selectivity for protein targets in cyclic molecules. The success of the restrained peptide format in cyclic conformation can help to develop novel targets with more and more favorable properties. In particular, regarding antimicrobial drugs, over 40 cyclic peptide drugs are, at present, in clinical use with promising preliminary results [1]. The majority of the cyclic antimicrobial drugs come from natural compounds and peptides/proteins as HBDs; the major advantage is that the increasing phenomenon of antibiotic resistance can be surmounted using natural compounds to fight bacterial and viral infections. In particular, HBDs analogs will retain several functions of the parental β-defensins that will confer a very potent antimicrobial action (Figure 3).

It is interesting to note that θ-defensins have been proposed as a scaffold to target protein-protein interaction in order to design novel therapeutics of interest for the pharmaceutical industry [74]. Conibear et al., has proposed that θ-defensins constitute a pseudo-cyclic cysteine ladder that can be used as a platform for two peptide epitopes with the same or different activities [74]. Similar to θ-defensins, cyclotides are disulfide-rich defense peptides from plants that have a head-to-tail cyclic backbone and cystine knot arrangement of three conserved disulfide bonds; this combination of the cystine knot motif and cyclic back-bone is referred to as a cyclic cystine knot (CCK) and is responsible for the exceptional stability of cyclotides [75,76]. Additionally, the exceptional stability of cyclotides has been exploited to generate novel therapeutics targeting protein-protein interactions [75,76].

It is now possible to rationally design novel cyclic or pseudo-cyclic antimicrobial peptides with high specificity towards microorganisms and minimal toxicity to eukaryotic cells. Future studies will lead to developing novel drugs that will help to fight antibiotic-resistant infections.

## Figures and Tables

**Table 1:** Cyclic peptide antibacterial drugs approved by the Food and Drug Administration (FDA).

Year of Approval	Generic Name	Indication	Mode of Action	Route of Administration	Company
2009	Telavancin	Skin and skin structure infections, nosocomial pneumonia	Bacterial cell-wall synthesis inhibitor	IV infusion	Theravance
2014	Dalbavancin	Skin and skin structure infections	Bacterial cell-wall synthesis inhibitor	IV infusion	Durata Therapeutics/Teva
2014	Oritavancin	Skin and skin structure infections	Bacterial cell-wall synthesis inhibitor	IV infusion	The Medicines Company
2006	Anidula fungin	Fungal infections	Fungal 1,3-β-d-glucan synthase inhibitor	IV infusion	Vicuron/Pfizer
Phase 2	POL7080	*P. aeruginosa* infections, Gram-negative infections	LptD protein homolog inhibitor, inhibits outer-membrane biogenesis		Polyphor
